# Time toxicity associated with treatment of metastatic or unresectable gastro-oesophageal cancers in the second-line setting

**DOI:** 10.1007/s00520-026-10359-w

**Published:** 2026-01-29

**Authors:** Rebecca Han Nguyen, Joanne Tang, Udit Nindra, Aflah Roohullah, Robert Yoon, Annette Tognela, Stephanie Hui-Su Lim, Ray Asghari, Wei Chua, Weng Ng

**Affiliations:** 1 Department of Medical Oncology, Liverpool Cancer Therapy Centre, Liverpool Hospital, Cnr Elizabeth & Goulburn Street, Liverpool, NSW Australia; 2 School of Clinical Medicine, South Western Sydney Clinical Campuses, UNSW Sydney, Sydney, NSW Australia; 3 School of Medicine, Western Sydney University, Sydney, NSW Australia; 4 Department of Medical Oncology, Macarthur Cancer Therapy Centre, Campbelltown Hospital, Campbelltown, NSW Australia; 5 Department of Medical Oncology, Bankstown Cancer Centre, Bankstown-Lidcombe Hospital, Bankstown, NSW Australia; 6 Ingham Institute for Applied Medical Research, Liverpool, NSW Australia

**Keywords:** Time toxicity, Gastric cancer, Oesophageal cancer, Shared decision making, Risk versus benefit

## Abstract

**Purpose:**

Patients with metastatic or unresectable gastro-oesophageal cancers (mGECs) have poor prognoses and often face high symptom burdens and rates of disease-related complications. Second-line treatments offer modest survival gains, which need to be balanced with treatment toxicities. Time toxicity (TT) is increasingly recognised as a hidden toxicity of cancer therapy, and thus, this study aimed to quantify TT to patients undergoing second-line treatment for mGECs.

**Methods:**

This was a retrospective cohort study across three major hospitals in Sydney, Australia. Records were reviewed for all patients who received second-line systemic therapy for mGECs over 10 years. TT was defined as the number of days patients spent physically interacting with the healthcare system.

**Results:**

Eighty patients were identified, with the majority male (83%) and a median age of 64 years. The median time on second-line treatment was 2.4 months, and the median overall survival from the commencement of second-line treatment was 5.8 months. Patients spent a median 25% of days in physical contact with healthcare, of which 20% were planned encounters (e.g. clinic appointments, scheduled investigations, and treatment days). TT was lower in patients who remained on second-line treatment for more than 2 months versus those on treatment less than 2 months (29% vs. 23%, *p* < 0.001). One in eight patients died within 30 days of receiving second-line treatment.

**Conclusion:**

Patients on second-line treatment for mGEC spent 1 in 4 days in contact with healthcare, and 30-day mortality following systemic treatment was high. These findings may guide decisions and informed consent surrounding second-line treatment in mGECs.

## Introduction

Gastro-oesophageal cancers (GECs) represent a significant health burden globally, ranking among the most common malignancies worldwide [1]. Up to half of patients with newly diagnosed GECs will have unresectable or metastatic disease [2], and in patients treated with curative intent, disease recurrence including metastatic recurrences remains common [3–5]. Despite recent treatment advances, prognosis for patients with unresectable or metastatic GECs remains poor, and the survival benefit of systemic treatment in the second-line setting remains limited [6, 7].

The proportion of patients with metastatic GECs who received first-line treatment and proceed to second-line treatment varies significantly in real-world studies, ranging from approximately 23 to 47% [8–11]. In clinical trials, the overall survival (OS) benefit of second-line systemic treatment is only 6 to 9 weeks compared to best supportive care alone, with response rates of only 10–29% [6, 12–18]. Practices for second-line systemic treatment vary, and the choice of treatment depends on a number of factors including prior treatments, patient performance status, and tumour biology [4, 6, 7] and may include single-agent chemotherapy (docetaxel, paclitaxel, or irinotecan), fluorouracil-based combination regimes, immunotherapy, targeted therapies (including ramucirumab and trastuzumab), or clinical trials, though all of these can be associated with substantial toxicities [7, 19].

In addition to treatment-related toxicities, patients with metastatic GECs also face high symptom burden and rates of disease-related complications which may necessitate hospital admission [20, 21]. Obstructive complications, such as malignant dysphagia and gastric outlet obstruction, are frequently encountered in these patients; these not only contribute to symptom burden, but exacerbate malnutrition and cancer cachexia, and may require palliative interventions [22, 23]. Complications such as bleeding and recurrent malignant ascites are also common and frequently necessitate hospitalisation and invasive procedures [24].

Given the high symptom burden and poor prognosis of patients with metastatic or unresectable GECs, it is important for patients and clinicians to consider the impact of any systemic treatment on patients’ quality of life, particularly in the second-line setting and beyond. In addition to treatment side effects, there is growing recognition regarding the hidden toxicities of cancer therapy. These include financial, time, mental, and social toxicities [25], and are systemically underreported and not well researched. Time toxicity (TT) has recently become more topical, especially in settings where survival gains from treatment are modest [26]. TT was first defined by Gupta et al*.* as a composite measure of days with physical healthcare system contact, which includes clinic visits, infusions, blood tests, seeking urgent care for side effects, or hospitalisations, where each of these encounters effectively results in a full day loss for patients and their carers [26]. Due to the limited understanding regarding the TT burden that patients experience in the second-line GEC setting, a multicentre retrospective cohort study across three major hospitals in Sydney, Australia, was conducted.

## Methods

This retrospective observational cohort study included patients from three cancer therapy centres in South West Sydney Local Health District. Patients who received second-line systemic therapy for metastatic or unresectable oesophageal, gastro-oesophageal junction, or gastric cancers between January 2015 and April 2024 were included. We excluded patients who were enrolled on clinical trials for second-line treatment.

Data was retrieved from patient electronic medical record databases. We collected data on patient demographics (age, gender, ethnic background), primary tumour characteristics, best response to first-line treatment, second-line treatment details (including treatment received, start and end dates of treatment, number of cycles, best response to treatment, and reason for stopping treatment), third-line treatment details, and survival statistics.

We defined TT as a composite measure as previously defined by Gupta et al. [26]. Time toxic days were defined as any day in which the patient is in physical contact with the healthcare system. This was divided into planned and unplanned interactions. Planned interactions comprised of routine appointments with medical or radiation oncology, palliative care teams and allied health, routine intravenous infusions, infusor disconnects, routine pre-treatment blood tests, scheduled imaging, and peripherally inserted central catheter (PICC) line or Portacath flushes. Unplanned interactions included unplanned reviews or appointments in day centres (e.g. for symptom management, review of side effects, or blood transfusion), hospitalisations, emergency department consultations, or unplanned procedures (e.g. paracentesis). Additional blood work, such as to assess pre-treatment blood count recovery following an episode of neutropaenia, or unscheduled imaging, such as a CT pulmonary angiogram performed for concern of pulmonary emboli, was also classified as unplanned interactions.

TT was calculated from the first day of second-line systemic treatment to the last day of treatment, calculated as the date of the last dose plus the length of the cycle (e.g. for a regimen with two weekly cycles, the end date is 2 weeks after the final dose). This was chosen to account for the ongoing effects of the treatment during the cycle period. Accordingly, TT was expressed as a proportion of days during active second-line systemic therapy, calculated from treatment commencement to treatment cessation, and does not include healthcare contact which occurred after second-line treatment discontinuation.

The project was approved by the Sydney South West Local Health District Human Research Ethics Committee. Data analysis was performed using R 4.4.1 (R Foundation, Vienna, Austria). Continuous variables were summarised using medians and interquartile ranges (IQRs), while categorical variables were reported as frequencies and percentages. Predictors of TT were assessed using the Wilcoxon rank-sum test, which was chosen due to the non-normal distribution of TT data. A *p*-value of less than 0.05 was considered statistically significant. Kaplan-Meier analysis was performed for survival analysis.

## Results

### Baseline characteristics

A total of 274 patients with metastatic GEC were screened, and 80 patients were included. The median age at the time of commencing second-line treatment was 64 years (range 30 years), and the majority of patients were male (*n* = 66). The most common primary tumour site was gastric (*n* = 44), followed by gastro-oesophageal junction (*n* = 28) (Table 1).

### Treatment response and outcomes

The most common treatment regime used in the second line was irinotecan-based treatments (*n* = 52, 65%), including 26% (*n* = 21) who received single-agent irinotecan. This was followed by taxane-based treatments, which were received by 19% (*n* = 15) of patients. Three patients received trastuzumab in combination with chemotherapy in the second line; one patient was enrolled in a clinical trial where trastuzumab was not administered as part of the trial protocol, and two patients had received trastuzumab with first-line chemotherapy and, upon progression, continued on trastuzumab with a change in chemotherapy backbone.

Best response to treatment was partial response in 15% (*n* = 12) patients with 25% (*n* = 20) stable disease as best response, while 60% (*n* = 48) were non-responders to second-line treatment. The median number of cycles of treatment completed was 4 (range 1–37), and median time on second-line treatment was 2.4 months (range 0.4 months). Most patients discontinued treatment due to disease progression or death (*n* = 66, 83%); there were 9 discontinuations from patient/physician decision and 5 discontinuations due to toxicity.

**Fig. 1:**
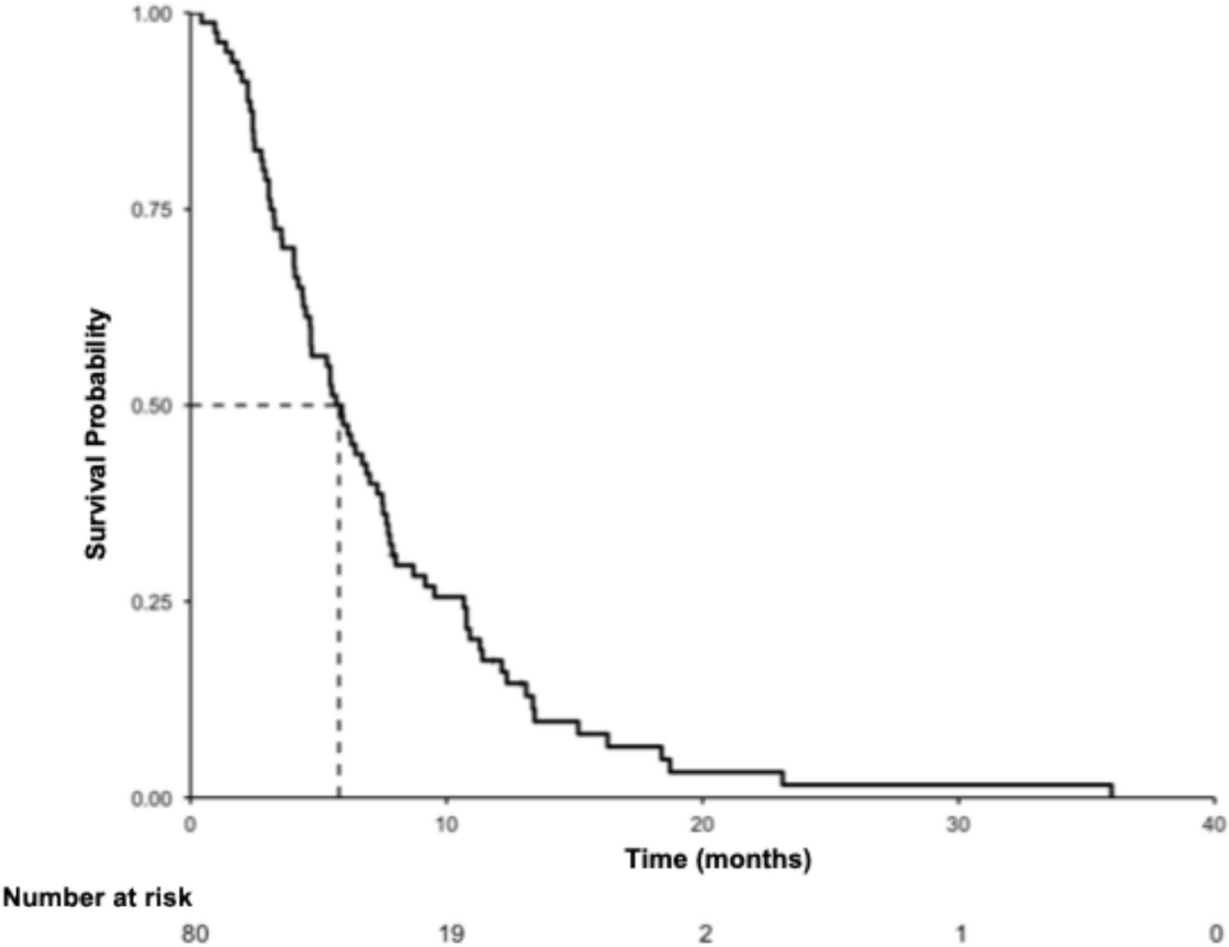
Overall survival from commencement of second-line treatment. Median OS 5.8 months (95% CI 4.7–7.5 months)

The median overall survival (OS), measured from commencement of second-line treatment, was 5.8 months (range 0.4 months) (Fig. 1). Median OS from commencement of first-line treatment was 14.1 months (4.2 months). Ten patients (12.5%) died within 30 days of receiving a dose of second-line treatment; there were six deaths due to disease progression, two deaths from an acute neurological event, one patient died due to an infective exacerbation of pulmonary fibrosis on chemotherapy, and the cause of death was not determined for one patient.

Twenty-five patients (31%) received third-line treatment; the most common third-line treatments were single-agent taxane chemotherapy (*n* = 10), clinical trial (*n* = 4), trifuridine/tiperacil (*n* = 3), and single-agent irinotecan (*n* = 3).

### Time toxicity

**Fig. 2:**
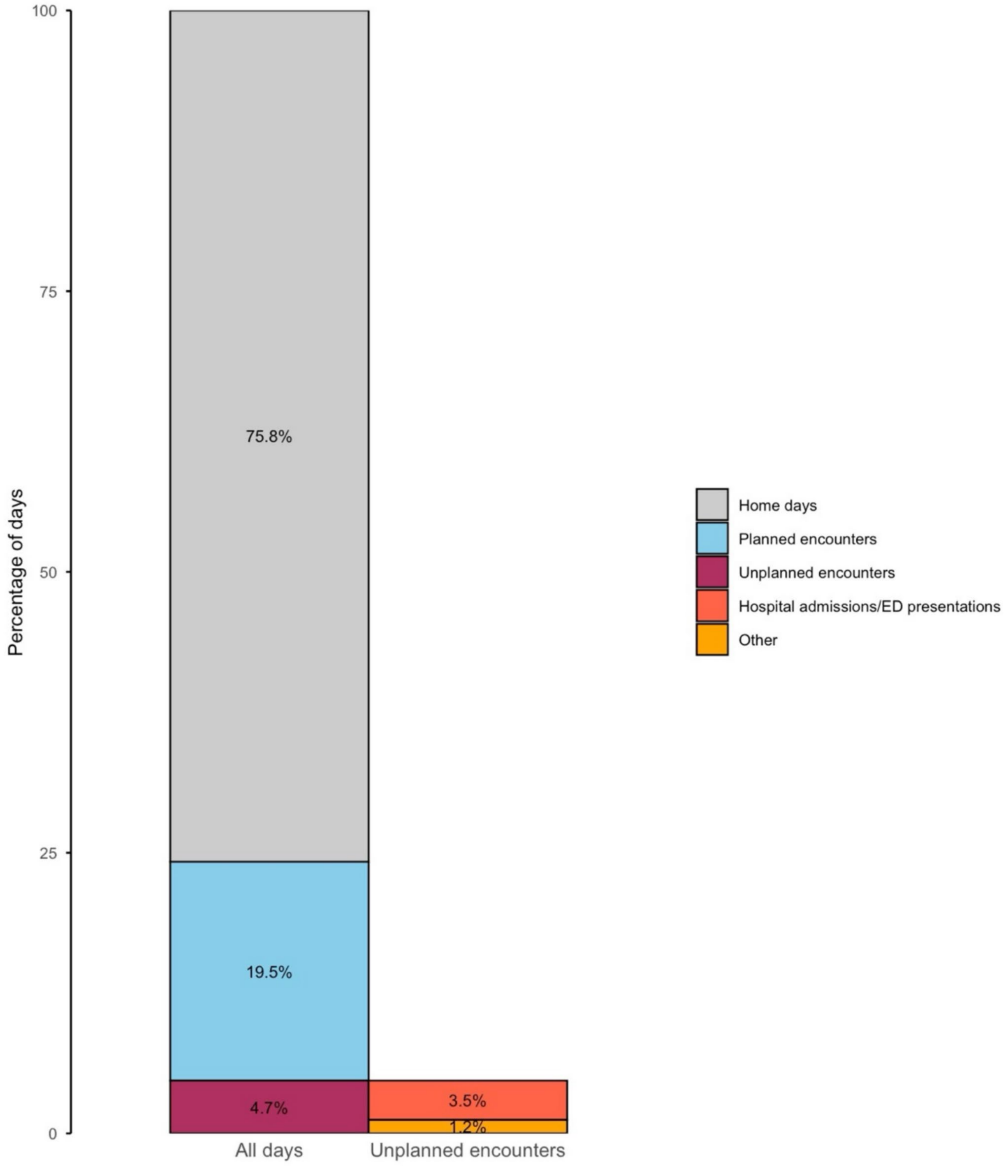
Summary of time toxicity experienced across all patients, expressed as the percentage of total days on second-line treatment

Across all patients, patients spent a median of 25% (range 9%–93%) of their days in direct contact with the healthcare system while on second-line treatment (Fig. 2). Planned encounters accounted for 19.5% of total days on treatment. In comparison, unplanned encounters contributed to 4.7% of total days, including 3.5% due to hospital admissions or emergency department encounters. Planned encounters were responsible for the majority of total TT in 86% of patients (*n* = 69).

In the entire cohort, there were a total of 76 hospital admissions or emergency department presentations while on second-line treatment, including 6 terminal admissions. Of the remaining presentations, the majority were secondary to malignancy-related complications (*n* = 42, 60%), followed by treatment-related complications (*n* = 20, 29%), and other (*n* = 8, 11%). Median length of stay for hospital admissions or emergency department presentations was 2.5 days (range 1–24 days).

The median proportion of time toxic days for patients who remained on second-line treatment for less than 2 months was 29%, compared to 23% for those on treatment for more than 2 months (*p* < 0.001). There were no significant differences in TT observed between other subgroups including patient age, gender, location of primary tumour, or HER2 status. When stratified by chemotherapy regime, there was no significant difference in time toxicity experienced by patients who received irinotecan-based treatment (median 25% of days on second-line treatment, IQR 22–29%) versus taxane-based treatment (median 25% of days on second-line treatment, IQR 21–29%).

## Discussion

To our knowledge, our retrospective cohort study is the first to assess the TT in patients receiving second-line systemic treatment for metastatic or unresectable GECs. In our cohort, median OS was under 6 months, and patients spent 25% of their days during active second-line systemic treatment in physical contact with the healthcare system. When excluding unplanned encounters, patients spent one in 5 days interacting with healthcare, representing the time burden associated with standard-of-care systemic treatment. This is in line with other studies which have assessed TT for patients undergoing standard-of-care treatment for advanced malignancies [23–25]. A Danish nationwide study for patients with metastatic lung cancer similarly found that these patients spent 22% of days in physical contact with healthcare following commencement of systemic treatment [27], while a retrospective study performed in Canada similarly found patients with metastatic non-small cell lung cancer receiving systemic treatment spent 22.6% of days in contact with healthcare, when measured from diagnosis to death [28]. In comparison, an American retrospective cohort study which assessed TT in patients with metastatic gastrointestinal malignancies (primarily consisting of patients with colorectal and pancreatic cancers) included over a third of patients who received supportive care only and found that from diagnosis to death, patients spent 25.8% of days with healthcare contact [29].

Our study excluded patients who were enrolled in clinical trials in the second-line setting, given the unique time investments required by clinical trial participation such as trial-mandated investigations and physical reviews [30]. In particular, early phase clinical trials (EPCTs) often have strict and rigorous patient schedules, given their primary focus is to demonstrate safety and determine dosages of novel treatments, and our previous study of TT among Australian EPCT participants found a higher proportion (29%) of time toxic days experienced by these patients [30].

We found that patients who continued on second-line treatment beyond 2 months had a lower proportion of time toxic days, compared to those who were treated for less than 2 months in the second line (29% versus 23%, *p* < 0.001). A cut-off of 2 months was chosen to reflect general clinical practice in our institutions where restaging scans are performed after 2 to 3 months of treatment in the palliative setting to assess treatment response. Hence, patients who are continuing treatment beyond 2 months are those who are having treatment responses with acceptable treatment tolerance. These patients are expected to have proportionally fewer time toxic days, which may be useful in informed decision-making with patients when deciding on whether they continue on palliative second-line treatment.

In our real-world cohort, median OS from commencement of second-line treatment was 5.8 months, which was similar to results from clinical trials. Irinotecan-based treatment was the most commonly prescribed regime in our cohort. Treatment with FOLFIRI has previously shown median OS of 6.2–6.7 months in second-line treatment of metastatic gastric or gastro-oesophageal junction cancers [13–15], while median OS with second-line single-agent irinotecan was 5.8–8.4 months [13, 16]. Treatment with single-agent taxane chemotherapy has similarly shown median OS of 5.2–9.5 months in patients who had progressed on first-line treatment [16, 17]. In our study, median TT during second-line therapy was similar for patients receiving irinotecan and taxane-based treatments, with both groups having a median of 25% of treatment days in healthcare contact. Framing these time demands alongside the modest survival gains demonstrated in clinical trials may be beneficial in shared decision-making with patients in balancing treatment benefit versus time burden of treatment.

In a small number of patients in our cohort, trastuzumab was administered in combination with chemotherapy in the second-line setting. This occurred for two reasons: one patient was enrolled in a clinical trial in the first-line setting and had no prior exposure to trastuzumab, while two patients continued on trastuzumab with a change in chemotherapy backbone following progression on first-line combination treatment with trastuzumab and chemotherapy. Although continuation of trastuzumab beyond first-line progression is not currently established in standard practice and is not supported by phase III trial evidence, this strategy has been explored by retrospective studies and a previous meta-analysis which suggested a possible progression-free survival benefit with this approach [31, 32]. While ramucirumab has previously shown OS benefits compared to placebo in patients with previously treated advanced gastric and gastro-oesophageal junction cancers [18], it is not currently funded for use in Australia and none of our patients received ramucirumab.

There were several limitations to our retrospective study. First, we did not capture healthcare encounters delivered at home, such as clinic appointments performed via telehealth, or home visits by the palliative care medical or nursing teams. Importantly, a subset of patients included in our study received treatment during the COVID-19 pandemic, whereby due to public health measures and risk management, some medical and allied health consultations were delivered via telehealth thereby reducing time spent in physical healthcare contact. As such, time toxicity reported in this cohort may underestimate what would be experienced with standard care. We were also unable to assess travel time to appointments, or the burden of visits to general practitioners, pharmacies, or other primary care providers, which could potentially underestimate the healthcare burden experienced by our patients. Further, we could not account for the time commitment required by carers for patients undergoing treatment.

Importantly, the study was not designed to assess TT experienced by patients who did not undergo systemic treatment after progression on first-line therapy, either due to patient preference or suitability for treatment. It is important to recognise that, as palliative systemic treatment can slow disease progression and provide symptom control, patients undergoing systemic treatment may therefore benefit from reduced hospitalisations for symptom management or complications such as obstruction or malignant ascites. Indeed, Gupta et al. had found that among patients with metastatic non-small cell lung cancer, the median percentage of days in contact with healthcare from diagnosis to death was 22.6% in those who received systemic treatment versus 42.4% in patients who received supportive care alone [28]. Hence, there is a need for further quantitative research into TT experienced by patients receiving best supportive care for advanced GECs, such as matched case-control studies, to further inform discussions regarding TT with treatment.

Nevertheless, this study provides a basis for discussion and shared decision making with patients when weighing up the benefits and toxicities of second-line treatment for advanced GECs, noting that modest overall survival benefits need to be balanced by frequent encounters with healthcare for patients and their carers while receiving treatment. Moreover, patients undergoing second-line therapy can also be informed that the burden of TT is with planned visits, and in our cohort, unplanned TT only accounted for 4.7% of total time spent by patients undergoing second-line therapies.

It is important to recognise that the term “time toxicity” implies that time spent with clinicians is inherently a negative experience, yet a substantial portion of time interacting with healthcare is essential in the delivery of systemic anti-cancer therapy, including treatment administration and clinical assessments to monitor for efficacy and toxicity. Without these interactions, patients would be unable to safely receive anti-cancer therapy or derive potential survival benefits from treatment, and hence, eliminating all time “toxicity” is neither feasible nor desirable. Furthermore, some patients may derive reassurance or improved symptom management from contact with their oncology team, and thus, higher documented TT may in itself be a positive experience for these patients or may help increase quality of life. Thus, in order to understand how TT can be improved, it is important to try and understand the impact healthcare visits have on patients’ health-related quality of life and where issues arise. Attempts to reduce TT by solely limiting healthcare visits or defaulting to a best supportive care approach may inadvertently reduce quality care, reduce patient support, or paradoxically increase healthcare burden for patients due to more frequent symptom-management presentations and complications related to progressive cancer.

## Conclusions

Our study is the first to review TT experienced by patients with metastatic GEC on second-line treatment. Patients spent 1 in 4 days in physical contact with the healthcare system while on treatment, and 30-day mortality following systemic second-line treatment was high (one in eight patients). Recognition of TT of treatment should feature during shared decision-making with patients when discussing second-line treatment for metastatic GECs.

**Table 1:** Patient demographics

	All patients (*n* = 80) *n* (%)
Age (years), median (range)	64 (30–83)
Male	66 (83)
Race/ethnicity	
Caucasian	55 (69)
East/Southeast Asian	9 (11)
Middle Eastern	7 (9)
South Asian	4 (5)
Pacific Islander	2 (3)
Hispanic	1 (1)
Other	2 (3)
Primary site of disease	
Gastric	44 (55)
Gastro-oesophageal junction	22 (28)
Oesophageal	14 (18)
Histopathology	
Adenocarcinoma	66 (83)
Squamous cell carcinoma	6 (8)
Signet ring	5 (6)
Small cell	3 (4)
HER2 status	
HER2 positive	10 (13)
HER2 negative	55 (69)
Not tested	15 (19)
Best response to first-line treatment	
Complete response	4 (5)
Partial response	37 (46)
Stable disease	22 (28)
Progressive disease	17 (21)
Second-line treatment	
Irinotecan-based	52 (65)
Taxane-based	15 (19)
Oxaliplatin-based	4 (5)
Trastuzumab + chemotherapy	3 (4)
Single agent immunotherapy	2 (3)
Other	4 (5)

## Data Availability

The datasets for this manuscript are not publicly available but requests to access the datasets should be directed to Rebecca Nguyen (RebeccaHan.Nguyen@health.nsw.gov.au).

## References

[1] Global Burden of Disease 2019 Cancer Collaboration**,** Kocarnik JM, Compton K, Dean FE, Fu W, Gaw BL, Harvey JD, Henrikson HJ, Lu D, Pennini A, Xu R et al ( 2022) Cancer incidence, mortality, years of life lost, years lived with disability, and disability-adjusted life years for 29 cancer groups from 2010 to 2019: a systematic analysis for the Global Burden of Disease Study 2019. JAMA Oncol 8(3):420–444. 10.1001/jamaoncol.2021.6987 10.1001/jamaoncol.2021.6987 PMC8719276 34967848

[2] Verstegen MHP, Harker M, van de Water C, van Dieren J, Hugen N, Nagtegaal ID, Rosman C, van der Post RS (2020) Metastatic pattern in esophageal and gastric cancer: influenced by site and histology. World J Gastroenterol 26(39):6037–6046. 10.3748/wjg.v26.i39.6037 33132653 10.3748/wjg.v26.i39.6037 PMC7584055

[3] Spolverato G, Ejaz A, Kim Y, Squires MH, Poultsides GA, Fields RC, Schmidt C, Weber SM, Votanopoulos K, Maithel SK, Pawlik TM (2014) Rates and patterns of recurrence after curative intent resection for gastric cancer: a United States multi-institutional analysis. J Am Coll Surg 219(4):664–675. 10.1016/j.jamcollsurg.2014.03.062 25154671 10.1016/j.jamcollsurg.2014.03.062

[4] Yang H, Wang F, Hallemeier CL, Lerut T, Fu J (2024) Oesophageal cancer. Lancet 404(10466):1991–2005. 10.1016/S0140-6736(24)02226-8 39550174 10.1016/S0140-6736(24)02226-8

[5] Mokadem I, Dijksterhuis WPM, van Putten M, Heuthorst L, de Vos-Geelen JM, Haj Mohammad N, Nieuwenhuijzen GAP, van Laarhoven HWM, Verhoeven RHA (2019) Recurrence after preoperative chemotherapy and surgery for gastric adenocarcinoma: a multicenter study. Gastric Cancer 22(6):1263–1273. 10.1007/s10120-019-00956-6 30949777 10.1007/s10120-019-00956-6 PMC6811385

[6] Smyth EC, Nilsson M, Grabsch HI, van Grieken NCT, Lordick F (2020) Gastric cancer. Lancet 396(10251):635–648. 10.1016/S0140-6736(20)31288-5 32861308 10.1016/S0140-6736(20)31288-5

[7] Ajani JA, D’Amico TA, Bentrem DJ, Cooke D, Corvera C, Das P, Enzinger PC, Enzler T, Farjah F, Gerdes H, Gibson M, Grierson P, Hofstetter WL, Ilson DH, Jalal S, Keswani RN, Kim S, Kleinberg LR, Klempner S, …, Pluchino LA (2023) Esophageal and esophagogastric junction cancers, version 2.2023, NCCN clinical practice guidelines in oncology. J Natl Compr Canc Netw. 21(4):393–422. 10.6004/jnccn.2023.0019 10.6004/jnccn.2023.0019 37015332

[8] Choi IS, Kim JH, Lee JH, Suh KJ, Lee JY, Kim JW, Kim SH, Kim JW, Lee JO, Kim YJ, Bang SM, Lee JS, Lee KW (2018) A population-based outcomes study of patients with metastatic gastric cancer receiving second-line chemotherapy: a nationwide health insurance database study. PLoS One 13(10):e0205853. 10.1371/journal.pone.0205853 30346970 10.1371/journal.pone.0205853 PMC6197657

[9] Dijksterhuis WPM, Verhoeven RHA, Pape M, Slingerland M, Haj Mohammad N, de Vos-Geelen J, Beerepoot LV, van Voorthuizen T, Creemers GJ, Lemmens VEPP, van Oijen MGH, van Laarhoven HWM (2020) Hospital volume and beyond first-line palliative systemic treatment in metastatic oesophagogastric adenocarcinoma: a population-based study. Eur J Cancer 139:107–118. 10.1016/j.ejca.2020.08.010 32980749 10.1016/j.ejca.2020.08.010

[10] Abraham P, Gricar J, Zhang Y, Shankaran V (2020) Real-world treatment patterns and outcomes in patients receiving second-line therapy for advanced/metastatic esophageal squamous cell carcinoma. Adv Ther 37(7):3392–3403. 10.1007/s12325-020-01394-y 32533533 10.1007/s12325-020-01394-y PMC7467430

[11] Luna J, Picker N, Wilke T, Lutz M, Hess J, Mörtl B, Xiong Y, Götze TO (2024) Real-world evidence of treatment patterns and survival of metastatic gastric cancer patients in Germany. BMC Cancer 24(1):462. 10.1186/s12885-024-12204-x 38614966 10.1186/s12885-024-12204-x PMC11016202

[12] Ter Veer E, Haj Mohammad N, van Valkenhoef G, Ngai LL, Mali RMA, van Oijen MGH, van Laarhoven HWM (2016) Second- and third-line systemic therapy in patients with advanced esophagogastric cancer: a systematic review of the literature. Cancer Metastasis Rev 35(3):439–456. 10.1007/s10555-016-9632-2 27417221 10.1007/s10555-016-9632-2 PMC5035657

[13] Sym SJ, Hong J, Park J, Cho EK, Lee JH, Park YH, Lee WK, Chung M, Kim HS, Park SH, Shin DB (2013) A randomized phase II study of biweekly irinotecan monotherapy or a combination of irinotecan plus 5-fluorouracil/leucovorin (mFOLFIRI) in patients with metastatic gastric adenocarcinoma refractory to or progressive after first-line chemotherapy. Cancer Chemother Pharmacol 71(2):481–488. 10.1007/s00280-012-2027-3 23192279 10.1007/s00280-012-2027-3

[14] Maugeri-Saccà M, Pizzuti L, Sergi D, Barba M, Belli F, Fattoruso S, Giannarelli D, Amodio A, Boggia S, Vici P, Di Lauro L (2013) FOLFIRI as a second-line therapy in patients with docetaxel-pretreated gastric cancer: a historical cohort. J Exp Clin Cancer Res 32(1):67. 10.1186/1756-9966-32-67 24330513 10.1186/1756-9966-32-67 PMC3850248

[15] Assersohn L, Brown G, Cunningham D, Ward C, Oates J, Waters JS, Hill ME, Norman AR (2004) Phase II study of irinotecan and 5-fluorouracil/leucovorin in patients with primary refractory or relapsed advanced oesophageal and gastric carcinoma. Ann Oncol 15(1):64–69. 10.1093/annonc/mdh007 14679122 10.1093/annonc/mdh007

[16] Hironaka S, Ueda S, Yasui H, Nishina T, Tsuda M, Tsumura T, Sugimoto N, Shimodaira H, Tokunaga S, Moriwaki T, Esaki T, Nagase M, Fujitani K, Yamaguchi K, Ura T, Hamamoto Y, Morita S, Okamoto I, Boku N, Hyodo I, WJOG 4007 Trial (2013) Randomized, open-label, phase III study comparing irinotecan with paclitaxel in patients with advanced gastric cancer without severe peritoneal metastasis after failure of prior combination chemotherapy using fluoropyrimidine plus platinum. J Clin Oncol 31(35):4438–4444. 10.1200/JCO.2012.48.5805 24190112 10.1200/JCO.2012.48.5805

[17] Ford HER, Marshall A, Bridgewater JA, Janowitz T, Coxon FY, Wadsley J, Mansoor W, Fyfe D, Madhusudan S, Middleton GW, Swinson D, Falk S, Chau I, Cunningham D, Kareclas P, Cook N, Blazeby JM, Dunn JA, COUGAR-02 Investigators (2014) Docetaxel versus active symptom control for refractory oesophagogastric adenocarcinoma (COUGAR-02): an open-label, phase 3 randomized controlled trial. Lancet Oncol 15(1):78–86. 10.1016/S1470-2045(13)70549-7 24332238 10.1016/S1470-2045(13)70549-7

[18] Fuchs CS, Tomasek J, Yong CJ, Dumitru F, Passalacqua R, Goswami C, Safran H, Dos Santos LV, Aprile G, Ferry DR, Melichar B, Tehfe M, Topuzov E, Zalcberg JR, Chau I, Campbell W, Sivanandan C, Pikiel J, Koshiji M, REGARD Trial Investigators (2014) Ramucirumab monotherapy for previously treated advanced gastric or gastro-oesophageal junction adenocarcinoma (REGARD): an international, randomized, multicentre, placebo-controlled, phase 3 trial. Lancet 383(9911):31–39. 10.1016/S0140-6736(13)61719-5 24094768 10.1016/S0140-6736(13)61719-5

[19] Ajani JA, D’Amico TA, Bentrem DJ, Chao J, Cooke D, Corvera C, Das P, Enzinger PC, Enzler T, Fanta P, Farjah F, Gerdes H, Gibson MK, Hochwald S, Hofstetter WL, Ilson DH, Keswani RN, Kim S, Kleinberg LR, Klempner Samuel J., Lacy Jill, Ly Quan P., Matkowskyj Kristina A., McNamara Michael, Mulcahy Mary F., Outlaw Darryl, Park Haeseong, Perry Kyle A., Pimiento Jose, Poultsides George A., Reznik Scott, Roses Robert E., Strong Vivian E., Su Stacey, Wang Hanlin L., Wiesner Georgia, Willett Christopher G., Yakoub Danny, Yoon Harry, McMillian Nicole, Pluchino LA (2022) Gastric cancer, version 2.2022, NCCN clinical practice guidelines in oncology. J Natl Compr Canc Netw 20(2):167–192. 10.6004/jnccn.2022.0008 35130500 10.6004/jnccn.2022.0008

[20] Pichel RC, Araújo A, Domingues VDS, Santos JN, Freire E, Mendes AS, Romão R, Araújo A (2022) Best supportive care of the patient with oesophageal cancer. Cancers 14(24):6268. https://doi.org/[insertDOI] 36551753 10.3390/cancers14246268 PMC9776873

[21] Merchant SJ, Brogly SB, Booth CM, Goldie C, Nanji S, Patel SV, Lajkosz K, Baxter NN (2019) Palliative care and symptom burden in the last year of life: a population-based study of patients with gastrointestinal cancer. Ann Surg Oncol 26(8):2336–2345. 10.3390/cancers14246268 30969388 10.1245/s10434-019-07320-z

[22] Enzinger PC, Mayer RJ (2003) Esophageal cancer. N Engl J Med 349(23):2241–2252. 10.1056/NEJMra035010 14657432 10.1056/NEJMra035010

[23] Mak M, Bell K, Ng W, Lee M (2017) Nutritional status, management and clinical outcomes in patients with esophageal and gastro-oesophageal cancers: a descriptive study. Nutr Diet 74(3):229–235. 10.1111/1747-0080.12306 28731604 10.1111/1747-0080.12306

[24] Harada K, Zhao M, Shanbhag N, Baba H, Ajani JA (2020) Palliative care for advanced gastric cancer. Expert Rev Anticancer Ther 20(7):575–580. 10.1080/14737140.2020.1781620 32543938 10.1080/14737140.2020.1781620 PMC7415645

[25] Bangs R (2024) Addressing the hidden toxicities of cancer: a call to action for clinicians, researchers and clinical trialists. BMJ Oncol 3(1):e000429. 10.1136/bmjonc-2024-000429 39886181 10.1136/bmjonc-2024-000429 PMC11235012

[26] Gupta A, Eisenhauer EA, Booth CM (2022) The time toxicity of cancer treatment. J Clin Oncol 40(15):1611–1615. 10.1200/JCO.21.02810 35235366 10.1200/JCO.21.02810

[27] Ording AG, Skjøth F, Poulsen LØ, Szejniuk WM, Jakobsen E, Christensen TD, Noble S, Overvad TF (2024) Time toxicity of systemic anticancer therapy for metastatic lung cancer in routine clinical practice: a nationwide cohort study. JCO Oncol Pract: OP2400526. 10.1200/OP-24-00526 10.1200/OP-24-00526 39705615

[28] Gupta A, Nguyen P, Kain D, Robinson AG, Kulkarni AA, Johnson DH, Presley CJ, Blaes AH, Rocque GB, Ganguli I, Booth CM, Hanna TP (2024) Trajectories of health care contact days for patients with stage IV non-small cell lung cancer. JAMA Netw Open 7(4):e244278. 10.1001/jamanetworkopen.2024.4278 38587847 10.1001/jamanetworkopen.2024.4278 PMC11002696

[29] Patel VR, Ramesh V, Tsai AK, Sedhom R, Westanmo AD, Blaes AH, Vogel RI, Parsons HM, Hanna TP, Ganguli I, Dusetzina SB, Rocque GB, Booth CM, Gupta A (2023) Health care contact days experienced by decedents with advanced GI cancer. JCO Oncol Pract 19(11):1031–1038. 10.1200/OP.23.00232 37738532 10.1200/OP.23.00232 PMC10667015

[30] Nindra U, Shivasabesan G, Childs S, Yoon R, Haider S, Hong M, Cooper A, Roohullah A, Wilkinson K, Pal A, Chua W (2023) Time toxicity associated with early phase clinical trial participation. ESMO Open 8(6):102046. 10.1016/j.esmoop.2023.102046 37979324 10.1016/j.esmoop.2023.102046 PMC10774969

[31] Palle J, Tougeron D, Pozet A, Soularue E, Artru P, Leroy F, Dubreuil O, Sarabi M, Williet N, Manfredi S, Martin-Babau J, Rebischung C, Abdelghani MB, Evesque L, Dreanic J, Hautefeuille V, Louafi S, Sefrioui D, Savinelli F, Mabro M, Rousseau B, Lecaille C, Bouché O, Louvet C, Lecomte T, Bonnetain F, Taieb J, Zaanan A (2017) Trastuzumab beyond progression in patients with HER2-positive advanced gastric adenocarcinoma: a multicenter AGEO study. Oncotarget 8(60):101383–101393. 10.18632/oncotarget.20711 29254172 10.18632/oncotarget.20711 PMC5731882

[32] Ter Veer E, van den Ende T, Creemers A, de Waal L, van Oijen MGH, van Laarhoven HWM (2018) Continuation of trastuzumab beyond progression in HER2-positive advanced esophagogastric cancer: a meta-analysis. Acta Oncol 57(12):1599–1604. 10.1080/0284186X.2018.1503421 30264641 10.1080/0284186X.2018.1503421

